# Association of American Medical Colleges

**Published:** 1911-04

**Authors:** 


### Association of American Medical Colleges

THE annual meeting of the Association of American Medical Colleges was held in Chicago, February 27 and 28, 1911. The president Dr. J. A. Witherspoon, of Vanderbilt University, Nashville, presided.

The following amendments to the constitution were adopted:

ARTICLE III: SECTION I.—Every college holding membership in this association shall, on and after January 1, 1912, require for matriculation a completed or unconditioned medical student's certificate, to be granted by a state medical examining and licensing board, or a board empowered by statute to grant such certificates, or a certificate of entrance to the academic department of any state university, or a certificate of entrance to an accredited university or college, providing that said certificate is granted on no less than the following requirements:

(a) A baccalaureate degree from an accredited college or university; (b) and (c) unchanged; (d and (e) eliminated.

SEC. 2. This examination must be conducted by or under the authority of the board of medical examiners of the state in which the college is located, or by a duly authorized examiner of the college entrance examination board, or the authorized examiner of an accredited university, state or otherwise, or by an examiner whose certificates are accepted by accredited colleges or universities, or by a method approved by the judicial council of this association.

SEC. 3. The term "accredited" as applied to high schools, academies, colleges and universities means institutions of that type that have been investigated and are accredited by the State University of their respective states, or by the North Central Association of Colleges and Secondary Schools, the Association of Colleges and Preparatory Schools of the Southern States, the Association of Colleges and Preparatory Schools of the Middle States and Maryland, the New England College Entrance Certificate Board, the Association of American Universities and the Association of State Universities, provided that such accrediting is based on Article III, Section 1, of this constitution.

SEC. 4. Colleges in membership in this association may honor the official credentials presented by students from other colleges having the standard requirements maintained by members of this association, excepting for the fourth year of their course, but no member of this association shall admit a student to advanced standing without first communicating with the College from which such student desires to withdraw, and receive from the dean, secretary or registrar of such college a direct written communication certifying to the applicant's standing. Credit for time or scholarship cannot be given beyond that of the college issuing the credentials, except by mutual agreement between the colleges.

SECS. 5, 6 and 7. Unchanged.

SEC. 8. Each student shall be obliged to attend not less than 80 per cent. of the exercises in every annual course of study for which he seeks credit. No student shall be given credit on examination unless he attains a grade of at least 70 per cent. or its equivalent in any other marking system. And no student shall be graduated unless he shall have attained a passing grade in each and all subjects of the required curriculum.

ARTICLE V: SEC. 1.—The entire course of four years shall consist of at least 4,000 hours for each student, and shall be grouped in divisions and subdivided into subjects; each division and subject to be allotted a certain number of hours.

Division 1, anatomy, 720 hours (18 per cent.); Division 2, physiology and chemistry, 600 hours (15 per cent.); Division 3, pathology, bacteriology and hygiene, 450 hours (11.25 per cent.); Division 4, pharmacology, materia medica and therapeutics, 240 hours (6 per cent.); Division 5, medicine and medical specialties, 970 hours (24.25 per cent.); Division 6, surgery and surgical specialties, 720 hours (18 per cent.); Division 7, obstetrics and gynecology, 300 hours (7.5 per cent.).

Colleges may reduce the number of hours in any subject not more than 20 per cent., provided that the total number of hours in a division is not reduced. Where the teaching conditions in a college are best subserved, the subject may be, for teaching purposes, transferred from one division to another. When didactic and laboratory hours are specified in any subject laboratory hours may be substituted for didactic hours.

SEC. 2. Each college in membership in this association shall print in every annual catalog or announcement a table of the total number of hours' work given in said college arranged both by subjects and years.

SEC. 3. Each college in membership in this association shall print annually a list of its students by classes.

ARTICLE VIII: SEC. 1—The stated meetings of this association shall occur annually at such place as the association may designate by vote, the time of meeting to be set by the officers and judicial council of the association.

(*To be continued.*)

